# AlphaML: A clear, legible, explainable, transparent, and elucidative binary classification platform for tabular data

**DOI:** 10.1016/j.patter.2023.100897

**Published:** 2023-12-13

**Authors:** Ahmad Nasimian, Saleena Younus, Özge Tatli, Emma U. Hammarlund, Kenneth J. Pienta, Lars Rönnstrand, Julhash U. Kazi

**Affiliations:** 1 Division of Translational Cancer Research, Department of Laboratory Medicine, Lund University, Lund, Sweden; 2 Lund Stem Cell Center, Lund University, Lund, Sweden; 3 Lund University Cancer Centre (LUCC), Lund University, Lund, Sweden; 4 Tissue Development and Evolution (TiDE), Department of Experimental Medical Sciences, Lund University, Lund, Sweden; 5 The Cancer Ecology Center, Brady Urological Institute, Johns Hopkins School of Medicine, Baltimore, MD, USA; 6 Department of Hematology, Oncology and Radiation Physics, Skåne University Hospital, Lund, Sweden

**Keywords:** machine learning, deep tabular learning, ensemble learning, precision medicine, feature selection, hyperparameter optimization, explainable AI, XGBoost, TabNet, drug sensitivity prediction

## Abstract

Leveraging the potential of machine learning and recognizing the broad applications of binary classification, it becomes essential to develop platforms that are not only powerful but also transparent, interpretable, and user friendly. We introduce alphaML, a user-friendly platform that provides clear, legible, explainable, transparent, and elucidative (CLETE) binary classification models with comprehensive customization options. AlphaML offers feature selection, hyperparameter search, sampling, and normalization methods, along with 15 machine learning algorithms with global and local interpretation. We have integrated a custom metric for hyperparameter search that considers both training and validation scores, safeguarding against under- or overfitting. Additionally, we employ the NegLog2RMSL scoring method, which uses both training and test scores for a thorough model evaluation. The platform has been tested using datasets from multiple domains and offers a graphical interface, removing the need for programming expertise. Consequently, alphaML exhibits versatility, demonstrating promising applicability across a broad spectrum of tabular data configurations.

## Highlights

• Introduced alphaML, a platform tailored for binary classification
• Supports a diverse array of classification algorithms with hyperparameter tuning
• Integrated custom metrics strategically address both underfitting and overfitting
• A versatile, user-friendly platform complemented by an intuitive graphical interface

## The bigger picture

In today’s data-driven landscape, the challenge extends beyond just building robust machine learning models; it encompasses ensuring their transparency and accessibility to a diverse audience. This challenge can be dissected into two critical aspects: first, crafting machine learning models that possess both robustness and adaptability, allowing them to handle diverse and ever-changing datasets effectively. Secondly, creating models that are transparent, interpretable, and user friendly, serving both seasoned data science veterans and newcomers alike. Building platforms that comprehensively address these concerns is important to bridge the gap between complex algorithms and clear interpretability, while also ensuring adaptability across diverse datasets.

## Graphical abstract

**Figure:**
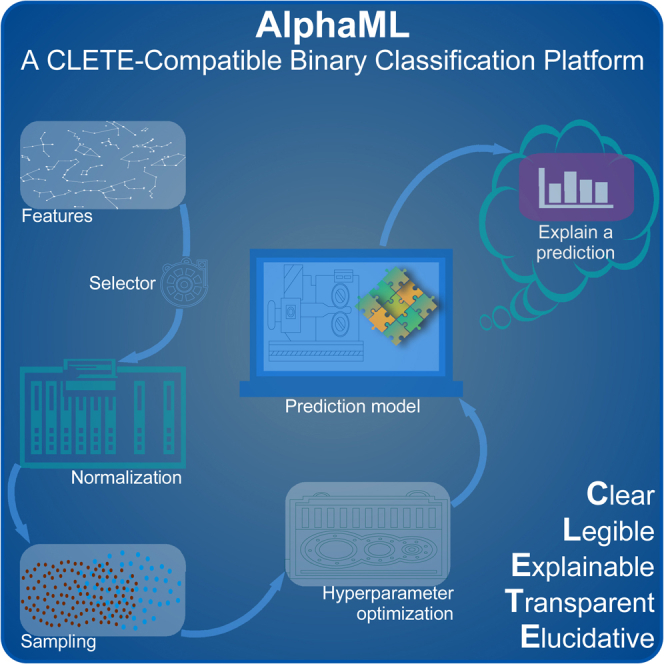

## Teaser

In an era of growing data complexity, the demand for sophisticated yet understandable tools in areas like health care and finance is intensifying. The alphaML platform, introduced here, delivers binary classification models that are powerful, transparent, and user friendly. Supporting a range of feature selection options, algorithms, and interpretative tools, it balances predictive accuracy with transparency. The platform has been tested across various domain-specific datasets and provides a user-friendly graphical user interface for non-expert users.

## Introduction

Machine learning algorithms have been extensively deployed in the scientific domain to establish correlations between biological attributes and drug responsiveness, particularly in experimental and preclinical settings.^1^^,^^2^^,^^3^^,^^4^ Most of the studies focusing on drug sensitivity prediction have relied on a standard suite of features to forecast the reactivity of a comprehensive range of therapeutics across various disease conditions. Although these studies establish a broad analytical framework, they often lack the precision and power necessary for clinical applicability. The reactivity of specific pharmaceutical compounds is contingent on an exclusive array of biological phenomena, which must be incorporated into the feature set for effective prediction specific to both drug and disease conditions. Recent research endeavors utilizing disease-centric and drug-specific biological attributes have exhibited notable predictive efficacy.^4^^,^^5^^,^^6^^,^^7^^,^^8^ Nevertheless, these methodological approaches necessitate substantial investment in terms of time and resources and demand a synergetic collaboration between researchers in the fields of biology and data science to ensure the effectiveness and rationality of the strategy.

The process of predicting drug sensitivity incorporates a multitude of computational procedures, encompassing aspects such as data preprocessing, feature extraction, data partitioning and sampling, optimization of model hyperparameters, and the employment of suitable machine learning algorithms.^2^ Moreover, comprehensive performance testing and interpretability evaluation are imperative for any algorithm after the training phase to elucidate the underpinning mechanism of its predictions. Although pharmacogenomic data generated within a laboratory environment constitute the core of the predictive model, the execution of data processing and model construction poses considerable challenges due to the intricate nature of the computational steps' integration, requiring proficiency in a high-level programming language such as Python or R.

Aside from the inherent intricacies involved in the formulation of a machine learning model, the regulation of overfitting and underfitting continues to pose significant hurdles across various machine learning methodologies. A multitude of hyperparameter optimization algorithms predominantly target the maximal efficacy of a machine learning model, which may not manifest an equivalent predictive capacity on test instances. This phenomenon arises as a result of hyperparameter optimization algorithms directing their focus on the optimal output of the predictive model, while neglecting to factor in discrepancies in performance between training and validation datasets.

In the present methodological outline, we detail the design and functionality of a machine learning system tailored to suit the needs of experimental biologists lacking the necessary temporal resources to acquire proficiency in computational languages such as Python or R. We’ve engineered unique performance assessment metrics, taking into account both the efficacy of the model and the disparity between training and validation accuracy, thereby ensuring automated regulation of overfitting and underfitting instances. The core concept introduced herein is denoted as CLETE (clear, legible, explainable, transparent, and elucidative), which encapsulates the system’s primary attributes—providing a lucid, thoroughly interpretable machine learning model. Additionally, the platform offers graphical user interfaces for both model creation and prediction functionality.

## Results

### The structure of alphaML platform

**Figure 1:**
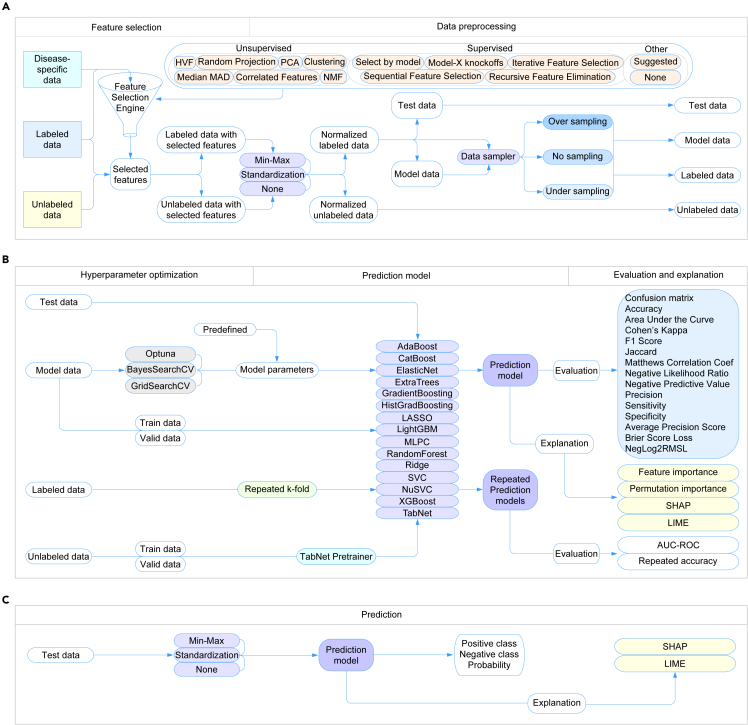
Schematic representation of the alphaML platform (A) The data management process. This illustrates the process of data accessibility verification in a specific directory of local storage. It also details the process of feature selection and preprocessing of retrieved data from local storage for model generation. The flowchart signifies how the system retrieves example datasets from FigShare in the absence of local data. (B) Model development and evaluation. This figure visualizes the subsequent stages following data preprocessing. It showcases the use of a data subset for hyperparameter optimization and the forwarding of optimized parameters to the model for its further evolution. It also exhibits how the system tests the model’s performance using the test dataset. (C) Prediction generation. This figure depicts the functionality of an integrated module dedicated to generating predictions using pre-established models. This highlights the platform’s ability to utilize already developed models for prediction tasks, emphasizing its flexibility and adaptability. A similar approach has been described recently.^7^

The core of alphaML machine learning is composed of various modules including data management, feature selection, data normalization, data sampling, hyperparameter tuning, model training and validation, testing, and model interpretability, reflecting a similar approach we have recently embraced.^7^ The data management module initially verifies the data accessibility in a particular directory of the local storage. If absent, it retrieves example datasets from FigShare.^9^ The retrieved data from the local storage is then utilized to select distinct features and preprocessed for model generation (Figure 1A). While not strictly essential, for pharmacogenomic datasets, such as those derived from microarray or RNA-seq platforms, we advocate for the submission of normalized and log2-transformed data. The iDEP online platform encompasses numerous data normalization options, including edgeR,^10^^,^^11^ which is instrumental in transforming raw counts. Subsequent to the preprocessing phase, a subset of data is used for hyperparameter optimization, and the optimized parameters are then forwarded to the model for subsequent evolution and performance testing using the test dataset (Figure 1B). Additionally, a distinct module has been integrated for generating predictions utilizing pre-established models (Figure 1C).

### The feature-selection module

The alphaML platform is tailored to either accept predefined feature lists or to carry out unsupervised or supervised feature selection strategies. The unsupervised strategies include identifying highly variable features (HVFs), using the median of median absolute deviation (MedianMAD), principal-component analysis (SelByPCA), random projection (RandomProjection), clustering (SelByClustering), and non-negative matrix factorization (SelByNMF). The supervised feature selection techniques include model-based selection (SelectByRF), recursive feature elimination (RecursiveFeatElim), sequential feature elimination (SeqFeatSel), and model-X knockoffs (ModelX). The system is further equipped with utilities for eliminating features demonstrating correlation exceeding 90% (“RemoveHighCorrFeat”) and iterative feature selection operations (IterativeFeatSel)—though computationally intensive—for pinpointing the top 1–3 features. The module integrates four consecutive feature selection alternatives, enabling the alphaML to conduct feature selection through diverse techniques. To exemplify, features with high variability can be identified via HVF or Median of MAD, facilitating an initial reduction in feature count prior to the execution of other techniques. Subsequent application of RandomProjection, PCA, NMF, or model-based selection can further condense the feature set prior to initiating resource-demanding supervised methodologies. In circumstances where feature selection is the sole requirement, the system’s feature selection module can function independently by opting for the algorithm “None.”

**Figure 2:**
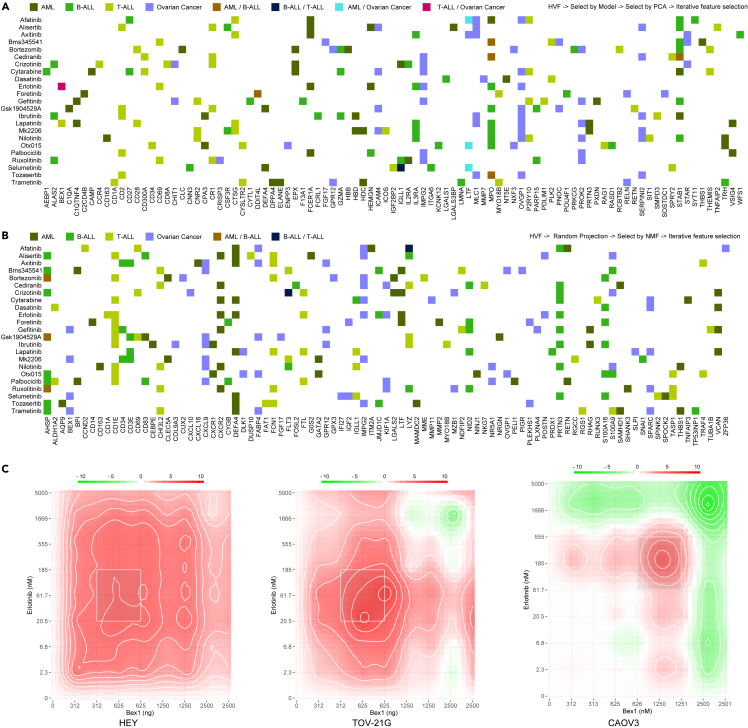
Sequential application of feature selection methods (A) The sequential implementation of the highly variable features (HVFs), model-based selection (Random Forest), principal-component analysis (PCA)-based selection, and iterative feature selection applied to AML, B-ALL, T-ALL, and ovarian cancer. (B) The sequential application of HVF, random projection, non-negative matrix factorization (NMF)-based selection, and iterative feature selection for the same diseases. A panel of 23 pharmaceutical compounds was assessed. The heatmap provides an overview of the feature selection procedures employed to assess the functionality of the feature selection module. (C) The synergistic effects of BEX1 overexpression and erlotinib treatment were quantitatively evaluated in ovarian cancer cell lines following a 48-h treatment period. DECREASE^42^ was used to expand combination response and SynergyFinder^43^ was used to calculate the synergy.

To assess the functionality of the feature selection module, we utilized four distinct pathological conditions as exemplars: acute myeloid leukemia (AML), B-cell acute lymphoblastic leukemia (B-ALL), T cell acute lymphoblastic leukemia (T-ALL), and ovarian cancer. We consecutively deployed four feature selection strategies in two unique sequences: HVF -> model-based selection -> PCA-based selection -> iterative feature selection (Figure 2A), and HVF -> Random Projection -> NMF-based selection -> iterative feature selection (Figure 2B) for 23 distinct pharmaceutical compounds. Our findings revealed that features displayed a significant degree of specificity related to the pathological condition, in addition to exhibiting a level of specificity for the pharmaceutical inhibitor and feature selection methodology employed. These observations underscore the necessity for a disease and drug-specific predictive model rather than a generalized model applicable to a broad spectrum of diseases and drugs. Subsequently, we discerned an amplified synergistic outcome when Brain Expressed X-linked protein 1 (BEX1) was subject to overexpression in ovarian carcinoma cellular cultures in conjunction with erlotinib administration (Figure 2C). This reinforces the concept that methodological selection for phenotypic features possesses significant utility in the elucidation of molecular interplay, thereby facilitating the derivation of efficacious therapeutic combinations.

### Data normalization module

In the realm of machine learning, the normalization of data is a critical preparatory stage that ensures equable contribution from disparate features toward the efficacy of the model, thereby augmenting the stability and efficiency of learning algorithms. In the absence of normalization, attributes exhibiting larger numerical spans may overshadow those with smaller numerical spans, potentially inducing biased model predictions. Techniques such as Min-Max scaling and Standardization are instrumental in alleviating these issues. Min-Max scaling, colloquially termed normalization, applies a transformation that coerces the data to conform to a specified range, customarily [0,1]. This is accomplished by deducting the minimum value of an attribute followed by division by the range of that attribute. This approach is advantageous when the dataset’s approximate boundaries are known, and the maintenance of precise distances between data points is nonessential. Contrastingly, Standardization, also recognized as *Z* score normalization, recenters the data about the mean, culminating in a zero mean, and scales it according to the standard deviation. Consequently, the ensuing distribution possesses a standard deviation of 1. This method is relatively impervious to outliers and is typically employed when the algorithm necessitates the supposition of normally distributed data, as seen in methods involving linear regression, logistic regression, and support vector machines. Min-Max scaling and Standardization methodologies both possess their merits and applications, and the preference for one over the other relies on the specific attributes of the data and the requisites of the machine learning algorithm in use. Through the transformation of the feature space to render it more conducive to the algorithms' presuppositions, data normalization serves as a critical determinant in the successful deployment of machine learning models. Moreover, beyond Min-Max and Standardization, alphaML platform allows an option “None” to forgo any form of transformation.

### Data sampling module

Within the domain of machine learning, specifically in relation to classification issues, techniques such as undersampling and oversampling are applied to rectify the issue of class imbalance. Class imbalance is a phenomenon where certain classes in the dataset are not as well represented as others. This disparity can create a biased model training process, with the model demonstrating superior ability in predicting the majority class and displaying subpar performance when dealing with the minority class. Undersampling mitigates this problem by diminishing the size of the majority class, thereby promoting a more equitable distribution among classes. This method entails the logical or random removal of instances from the majority class with the aim of aligning the sample size with that of the minority class. Though undersampling can effectively balance class distribution, it poses the risk of excluding potentially valuable data that could contribute significantly to model training. In contrast, oversampling attempts to rectify class imbalance by amplifying the number of occurrences within the minority class. This is typically accomplished by either replicating instances from the minority class or synthesizing new instances grounded in the extant data, the latter approach often employing a technique known as synthetic minority oversampling technique (SMOTE). Despite its efficacy in balancing class distribution, oversampling may cause model overfitting to the minority class due to the utilization of duplicated or synthesized instances.

**Figure 3:**
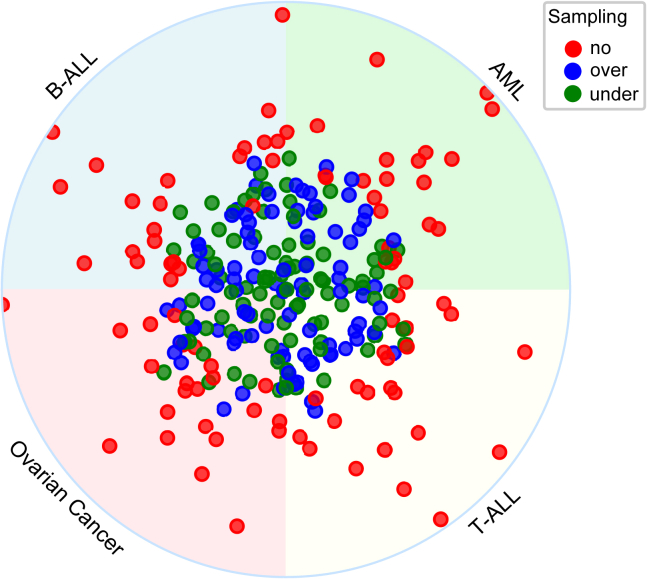
A comparative analysis of varied sampling techniques We employed an XGBoost classifier, fixed with predetermined parameters, to construct and evaluate binary classification models. Prior to implementing undersampling or oversampling procedures, the test samples were distinctly partitioned. The radial distance of each point from the circle’s origin corresponds to the normalized NegLog2RMSL for each inhibitor, where a higher value indicates superior performance.

The imbalanced-learn library was deployed in alphaML to regulate the application of undersampling and oversampling.^12^ Furthermore, the sampling module provides a “no sampling” method that maintains the original proportions of the major and minor classes. However, during the model construction phase, a class imbalance parameter is invoked to balance the ratio. Notably, test samples always remain unaltered by the sampling process. We ensure this by partitioning the test sample prior to its submission to the sampling module, thereby guaranteeing that no sampling biases afflict the test data. Through the examination of pharmacogenomic data from 23 inhibitors and four disease models, it was observed that the “no sampling” method consistently surpassed the performance of both oversampling and undersampling methods measured by the normalized NegLog2RMSL (Figure 3), intimating that the adoption of a sampling procedure may not invariably contribute toward enhancing the efficacy of a machine learning model. Nonetheless, this observation could exhibit high specificity to a particular problem, thus underscoring the perpetual necessity of a comprehensive comparative analysis.

### Hyperparameter optimization module

The optimization of hyperparameters constitutes a fundamental stage in the development of robust and high-precision machine learning models. Hyperparameters denote factors that are not inherently derived within predictors but rather are determined by the user preceding the model training process. Their influence on model performance is considerable as they preside over the intricacies of the model, the extent of regularization, and the learning rate, among other factors. We have assimilated three varied hyperparameter tuning methodologies into our process: grid search cross-validation (GridSearchCV), Bayesian optimization, and Optuna. GridSearchCV is a conventional technique employed in hyperparameter tuning. It conducts a comprehensive exploration over a manually prescribed subset within the hyperparameter space of a learning algorithm. For each hyperparameter combination, it trains a model and gauges its performance through cross-validation, ultimately selecting the hyperparameters that offer the highest performance metric. Although it provides a comprehensive and straightforward approach, GridSearchCV can be computationally burdensome, especially when dealing with numerous hyperparameters and extensive datasets. Bayesian optimization, implemented in libraries such as Scikit-Optimize, offers a more proficient method for hyperparameter tuning.^13^ It constructs a probabilistic model of the objective function, leveraging previous evaluation outcomes, and employs this model to choose the hyperparameters that exhibit the highest potential for evaluation. Hence, Bayesian optimization is particularly apt for optimization problems where function evaluations impose a significant computational cost. Lastly, Optuna presents another proficient framework for hyperparameter optimization, effectively automating the hyperparameter tuning process.^14^ It integrates several cutting-edge methods for efficacious hyperparameter exploration, such as handling categorical parameters, pruning unpromising trials, and automated concurrency management.

**Figure 4:**
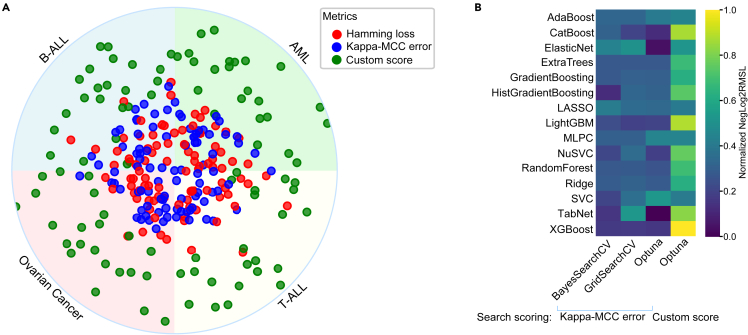
The "custom_score" exhibits superior performance in comparison with alternative scoring metrics (A) The hyperparameters for the XGBoost classifier were optimized utilizing Optuna, encompassing three distinct scoring methods. The model incorporated four distinct disease paradigms along with 23 inhibitors. Each individual dot denotes the normalized NegLog2RMSL value, plotted in reference to the origin of the circle considered as zero. (B) Diverse search methods, which integrated either "kappa_mcc_error" or "custom_score," were employed for the optimization of hyperparameters across a selection of algorithms. These methods were tested using T-ALL samples in conjunction with the trametinib inhibitor.

In assessing the efficacy of hyperparameter search, we executed the Optuna hyperparameter optimization protocol. Three performance evaluation indices were exploited: the Hamming loss that signifies the converse of accuracy, "kappa_mcc_error," and a uniquely devised metric, "custom_score." The Hamming loss and the "kappa_mcc_error" metrics utilize validation data to generate a numerical measure, consequently guiding the model toward its optimal performance aligned with the validation score. Contrarily, the "custom_score" considers both the training and validation datasets, aiming to attenuate the divergence between the performance on training and validation while simultaneously endorsing the model’s paramount performance. In accordance with data derived from 23 inhibitors and four disease models, the incorporation of "custom_score" within the Optuna scoring architecture seems to assure superior performance as measured by NegLog2RMSL (Figure 4A). Additionally, while juxtaposing different algorithms with various search methods, "custom_score" amalgamated with Optuna exhibited the highest NegLog2RMSL (Figure 4B). In aggregate, the evidence indicates that the employment of "custom_score" can augment the performance of binary classification models.

### Algorithms

**Figure 5:**
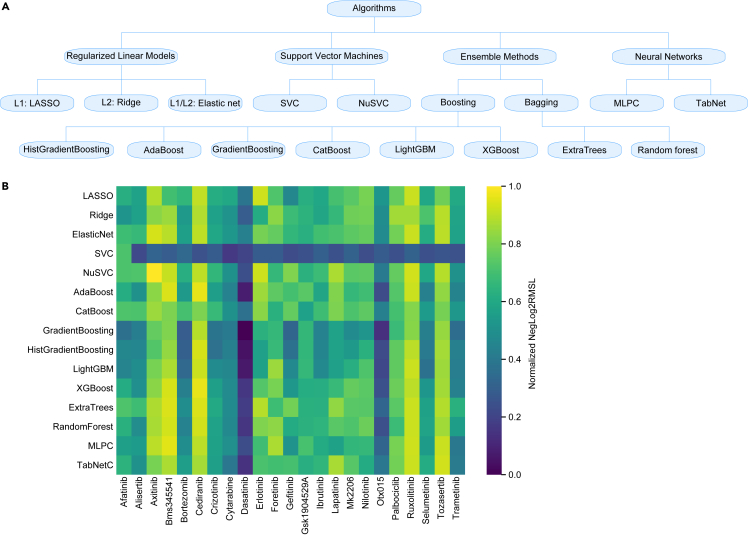
Comparison of different algorithms (A) A general classification of algorithms used in alphaML platform. (B) AML dataset was used to select features using HVF function. Hyperparameters were optimized using Optuna. The NegLog2RMSL scoring was used to select the best parameters.

The alphaML platform integrates 15 algorithms, which can be classified into four categories: regularized linear models, support vector machines, ensemble methods, and neural networks, as represented in Figure 5A. A brief description of each model is presented in the experimental procedures section. We conducted a comparative analysis of all algorithms using 23 inhibitors. The AML dataset was leveraged as a source of feature selection in this particular context. For hyperparameter optimization, we employed the Optuna framework and the “custom_score” to minimize the error. The comparative effectiveness of diverse algorithms and inhibitors was evaluated using the standard measure of normalized NegLog2RMSL. A subset of pharmacological compounds, namely axitinib, BMS-345541, cediranib, ruxolitinib, and tozasertib, demonstrated superior efficacy in most algorithmic interpretations (Figure 5B). While it was observed that ensemble methodologies consistently outperformed others in terms of normalized NegLog2RMSL, algorithms employing regularization techniques also yielded commendable outcomes, underlining the relevance of regularization in processing high-dimensional datasets.

### The interpretive module of the model

The preponderance of algorithmic configurations within the alphaML framework encompasses innate methodologies for deducing feature importance. However, certain algorithms, namely linear algorithms, SVCs, and multi-layer perceptron classifier (MLPC) are devoid of such integral functionality. Consequently, in addition to these inherent methodologies, we have assimilated permutation feature importance from sklearn and global feature importance from Shapley Additive Explanations (SHAP), which possess the capability of discerning influential features, independent of their algorithmic origins.^15^^,^^16^^,^^17^ SHAP is a cohesive measure of feature significance, attributing an importance value to each feature for a specific prediction. This measure finds its roots in the Shapley values concept, a derivative of cooperative game theory that ensures equitable distribution of payoffs among players depending on their contribution to the total payoff.^18^ SHAP values provide an interpretation of the influence of a specific value for a particular feature compared with the prediction we would procure if that feature assumed a baseline value. Essentially, SHAP attributes an importance value to each feature for a specific prediction, symbolizing the degree to which the feature’s value contributes to the deviation of the prediction from the baseline prediction. SHAP proposes a methodology to calculate universal feature significance by averaging the absolute SHAP values for each feature across all instances within a dataset. This provides an understanding of the effect of each feature on model prediction across numerous instances, as opposed to a singular instance. Features are then typically stratified by these universal importance values, providing a perspective on which features are predominantly influential in the model’s predictions on average. Hence, SHAP can be employed to discern both local and universal feature significance.

Additionally, we have assimilated the local interpretable model-agnostic explanations (LIME), a technique devised for elucidating the predictions of any machine learning classifier.^19^ LIME approximates the decision boundary of the complex model locally with a simplified model, such as a linear model, which is easier to interpret. For a specific instance, LIME generates a new dataset of perturbed samples, procures the predictions of these samples using the complex model, and then fits the simplified model on this new dataset. The weights of the simplified model are learned based on the proximity of each perturbed sample to the instance of interest. The coefficients of the simplified model serve as an explanation of the contribution of each feature to the prediction for the instance of interest. The advantage of LIME is that it offers local interpretability, elucidating the model’s decisions on a per-instance basis. However, it can also be employed to evaluate universal feature significance by aggregating local explanations for a representative set of instances.

**Figure 6:**
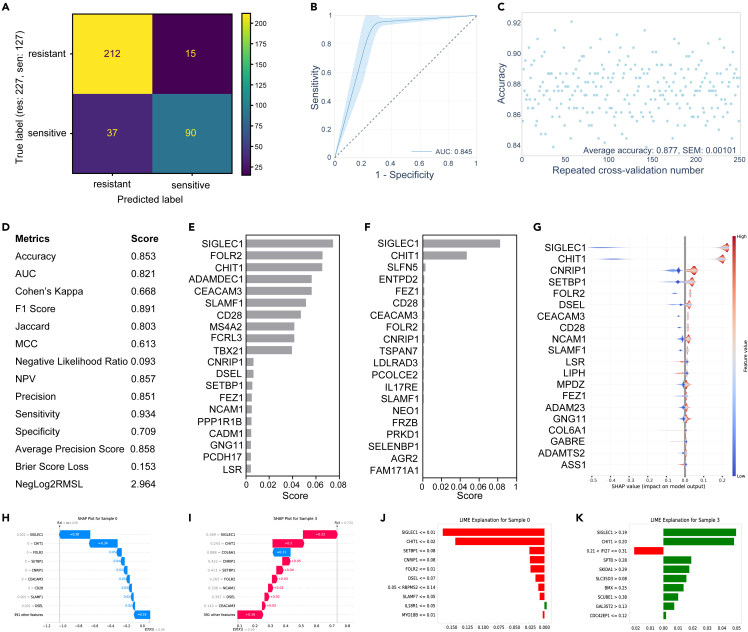
Evaluation and interpretation of predictive model The dataset composed of predictive and explanatory data derived from crizotinib and the XGBoost algorithm. (A) A confusion matrix was constructed utilizing the test sample dataset. (B) The ROC-AUC was generated through a 5-fold CV approach. (C) Average precision was ascertained via a 5-fold CV technique. (D) Various metric scores were computed utilizing the test sample data. (E) Feature significance was established using the inherent function of XGBoost, wherein the top 20 prominent features were illustrated. (F) A permutation feature importance method was employed to identify key features. (G) Global SHAP values were charted for the test samples. (H) An SHAP local explanation was provided for the sample predicted as sensitive. (I) An SHAP local explanation was presented for the sample predicted as resistant. (J) An explanation using LIME was provided for the sample anticipated as sensitive. (K) A LIME explanation was delineated for the sample expected to be resistant.

The evaluation of model efficacy was conducted through the application of test data and the implementation of a 5-fold repeated cross-validation. Figure 6 illustrates data derived specifically from the application of crizotinib and the XGBoost algorithm. The construction of the confusion matrix (Figure 6A) was achieved using test data, while the receiver operating characteristic area under the curve (ROC-AUC) (Figure 6B) and accuracy plot (Figure 6C) were generated through the use of 5-fold repeated cross-validation. Additionally, the computation of scores for different metrics (Figure 6D) was conducted for the test sample. The test samples were further utilized for the ascertainment of feature importance from the model (Figure 6E), the permutation (Figure 6F), and the SHAP (Figure 6G). Feature importance for individual test samples was depicted utilizing both SHAP and LIME, with exemplification through a crizotinib-sensitive sample (Figures 6H and 6J) and a crizotinib-resistant sample (Figures 6I and 6K). Interestingly, a recurring pattern was observed wherein the expression of SIGLEC1 and CHIT1 appeared to exert influence on the prediction outcomes.

### Prediction module

**Figure 7:**
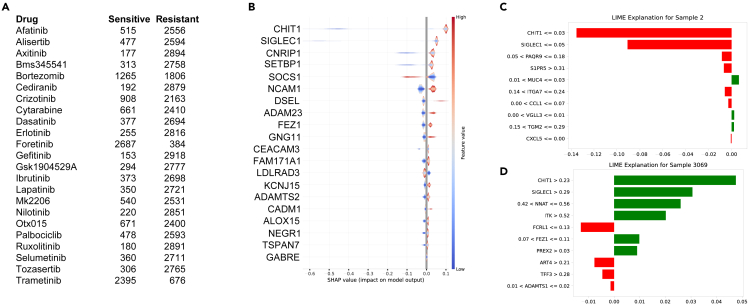
Inferential analysis with XGBoost algorithms AML patient dataset was employed to forecast the responsiveness toward 23 therapeutic drugs. (A) Enumeration of samples deemed sensitive or resistant as discerned by the XGBoost algorithm. (B) Comprehensive SHAP illustration associated with the crizotinib prediction. (C) LIME elucidation corresponding to the sample classified as sensitive. (D) LIME interpretation pertinent to the sample designated as resistant.

The prediction module integrated within the computational framework operates as an autonomous unit, maintaining independence from the model construction platform. This prognostic component accepts a trained algorithm and corresponding feature set utilized during the model training phase, enabling predictive operations and elucidation of the prediction rationale via SHAP and LIME, utilizing data as an input source. RNA-seq data from 3,071 AML patients were procured from the Genomic Data Commons (GDC) data portal (portal.gdc.cancer.gov) for this purpose. The samples demonstrated an amplified sensitivity toward bortezomib, a proteasome inhibitor, foretinib, a multi-targeted tyrosine kinase inhibitor, and trametinib, an MEK inhibitor (Figure 7A), thereby underscoring the potential of a predictive model in identifying patient-specific therapeutic strategies. In addition, the global feature significance as indicated by SHAP (Figure 7B) and local importance as defined by LIME (Figures 7C and 7D) provided valuable insights into influential regulators, with crizotinib serving as a prime example in this context.

### Versatility of the platform

**Figure 8:**
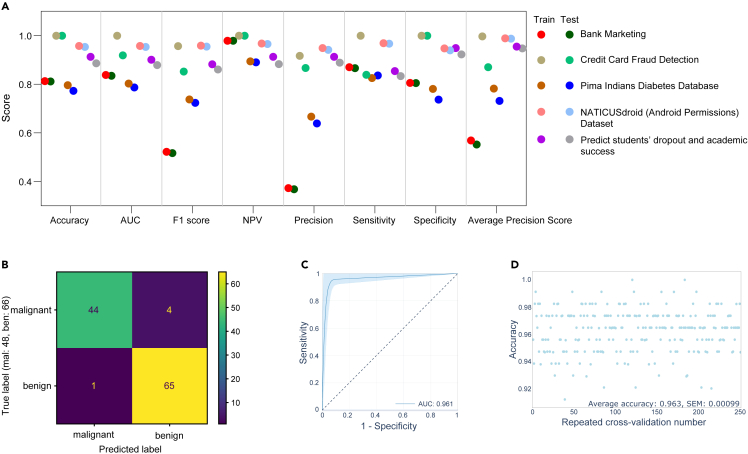
Evaluation of model adaptability across diverse datasets To assess the platform’s versatility, we tested it against six disparate datasets. (A) Performance metrics were calculated utilizing the XGBoost algorithm, with hyperparameter tuning executed through Optuna. All attributes encompassed in these datasets were considered. (B–D) Utilizing the Wisconsin Breast Cancer (Diagnostic) dataset, the model was constructed and validated employing the XGBoost framework, with Optuna facilitating hyperparameter optimization. See also Figures S1, S2 and S3.

To evaluate the robustness and applicability of our platform across diverse domains, we deployed it on six distinct datasets, namely: Bank Marketing, Credit Card Fraud Detection, Pima Indians Diabetes, NATICUSdroid (Android Permissions), Student Dropout and Academic Success prediction, and Breast Cancer Wisconsin (Diagnostic) dataset. The first five datasets were utilized to ascertain the consistency of the model’s predictive performance between the training and testing subsets, as implemented through the XGBoost algorithm (Figure 8A). For the final dataset—Breast Cancer Wisconsin (Diagnostic)—we utilized it to generate a confusion matrix (Figure 8B), ROC-AUC curve (Figure 8C), repeated accuracy plot (Figure 8D), SHAP plots (Figures S1 and S2), and LIME plots (Figure S3). The cumulative evidence affirms the broad-spectrum utility of our proposed platform.

## Discussion

In the presented research, we delineated a facile binary classification framework aimed at predicting therapeutic response in oncology, employing advanced machine learning techniques. This versatile platform integrates an assortment of critical components, such as feature selection, data normalization, data sampling, hyperparameter optimization, model training and validation, testing, and model interpretability. The assemblage of these modules enables a comprehensive machine learning solution. Its potency was validated by employing pharmacogenomic data across a broad spectrum of drugs and diseases, signifying its wide applicability to various tabular data formats.

In the past years several automated machine learning platforms have been developed, notably Auto-WEKA and Auto-Sklearn. The Auto-WEKA evaluates combinations of algorithms and their respective hyperparameters to find the best model for a given dataset.^20^ The initial version of Auto-Sklearn integrated a range of algorithms from the scikit-learn library, one of the most popular Python toolkits for machine learning. This version also included several feature selection approaches to assist in determining the most relevant attributes or columns in a dataset for predictive modeling.^21^ Over time, the developers sought to refine and enhance the platform. With the release of Auto-Sklearn 2.0, substantial modifications were made to the core algorithm, resulting in improved performance and efficiency.^22^ These modifications were not only in terms of hyperparameter optimization but also took into account meta-learning, which helps in recommending algorithms based on meta-features of the dataset. This newer iteration further solidified Auto-Sklearn’s position as a leading auto-ML tool, streamlining the machine learning pipeline by automatically configuring the best preprocessing, feature engineering, and algorithm selection strategies. The alphaML platform was designed with a distinct objective in mind. Unlike many auto-ML systems, alphaML does not strictly automate feature selection or algorithm determination. Instead, it offers users an intuitive interface that enables efficient customization, spanning feature selection, normalization, hyperparameter search, data imbalance control, algorithm selection, and model interpretation, all through a graphical user interface (GUI). This design philosophy sets alphaML apart from many prevalent auto-ML platforms. While there are overlaps in the algorithms and feature selection methods between alphaML and other auto-ML tools, our platform introduces several algorithms, including HVFs and Model X knockoffs for feature selection. We have also incorporated cutting-edge algorithms such as Catboost, LightGBM, XGBoost, and TabNet, which, as of now, have not been integrated into the scikit-learn platform. A recent study examining various algorithms for tabular data learning highlighted the superiority of several of these algorithms in diverse prediction tasks.^23^

The feature selection module is operable as an autonomous entity, and its efficacy was evaluated across a variety of disease classes utilizing 23 distinct pharmacological compounds. The results emphatically illustrate that the selected features manifest disease-specific as well as drug-specific traits, thereby inferring the indispensability of predictive models tailored to specific pathologies and pharmaceutical agents. Additionally, through the overexpression of BEX1 in ovarian cancer cells, partial validation of the utility of the feature selection component has been achieved.

The imbalanced-learn library was employed within the alphaML framework to govern undersampling and oversampling applications. Interestingly, the results indicated a preference for the “no sampling” approach over traditional methods in analyzing pharmacogenomic data. The observations suggest that preserving the inherent major and minor class proportions in the dataset yielded superior performance, even as the class imbalance parameter was triggered during the model’s construction phase. However, the research also recognizes that the comparative performance of these sampling methods could vary significantly based on the problem at hand, highlighting the constant requirement for comprehensive comparative assessments. Test data integrity was ensured via pre-sampling partitioning, thereby eliminating any potential sampling bias.

The Optuna hyperparameter optimization technique was employed to assess its efficacy, using three performance indices: Hamming loss, kappa_mcc_error, and a newly introduced metric dubbed “custom_score.” The Hamming loss and kappa_mcc_error indices use validation data to steer the model’s optimization, whereas the custom_score metric utilizes both training and validation datasets to bridge performance gaps. When integrated with the Optuna scoring framework, the custom_score metric appeared to augment performance, as indicated by the superior NegLog2RMSL metrics in the study of 23 inhibitors across four disease models. A comparative analysis of different algorithms and search methods revealed that combining custom_score with Optuna yielded the most favorable NegLog2RMSL, signifying that the incorporation of the custom_score metric may potentially enhance binary classification model performance. This research featured an exhaustive comparison of various algorithms using 23 inhibitors, sourcing feature selection from the AML dataset. The Optuna framework was employed for hyperparameter optimization, alongside a “custom_score” to curtail errors.

Normalized NegLog2RMSL was used as the benchmark for comparative performance assessment, revealing superior efficiency of certain drugs such as axitinib, BMS-345541, cediranib, ruxolitinib, and tozasertib. The results underscored the commendable performance of ensemble methods and the significance of regularization techniques when dealing with high-dimensional datasets. Model evaluation was performed using test data and 5-fold repeated cross-validation, as depicted in the data derived from the application of crizotinib and the XGBoost algorithm. Metrics such as the confusion matrix, ROC-AUC, accuracy plot, and various other metric scores were calculated and visualized, providing diverse insights into the model’s performance and efficacy. Additionally, test samples were used to elucidate the model’s feature importance, as illustrated by permutations and SHAP. The recurrent pattern of SIGLEC1 and CHIT1 expression impacting prediction outcomes was notable, thereby indicating potential predictive value of these biomarkers.

The prediction module, a standalone component of the computational framework, functions independently of the model construction platform. It ingests a pre-trained algorithm and corresponding feature set, thus enabling predictive functionality and the explanation of prediction rationales via SHAP and LIME, utilizing RNA-seq data from 3,071 AML patients procured from the GDC data portal. The sensitivity displayed by the patient samples toward bortezomib, foretinib, and trametinib underscores the potential of predictive models in devising patient-specific therapeutic strategies. Moreover, the global and local feature importance as delineated by SHAP and LIME, respectively, offer insights into influential regulators, exemplified by crizotinib.

In conclusion, this study lays the groundwork for a foundational framework that holds significant promise in optimizing therapeutic strategies for oncology patients. It emphasizes the need for further validation and exploration. By harnessing the power of integrated analysis of intricate pharmacogenomic data, our approach showcases the importance of custom-tailored predictive models for both disease-specific and drug-specific scenarios. Additionally, it underscores the pivotal role of computational methodologies in advancing the field of personalized medicine.

### Limitations of the study

While the alphaML platform boasts extensive functionality and versatility, there are certain operational specifics to consider. Users should place their input data in a designated folder, alphaML_data, located within the computer’s Documents directory. It is essential to adhere to the precise naming conventions for both the folder and files as detailed (Note S1). Should these conventions not be met, alphaML defaults to downloading sample files. Additionally, the platform currently accepts data solely in the CSV format.

The alphaML platform is currently designed to support only label encoding. For categorical data, users are advised to employ label encoders such as LabelEncoder or OneHotEncoder from sklearn.preprocessing. It is also worth noting that alphaML defaults to substituting NaN values with zeros, without attempting any imputation. For those who require data imputation, we suggest utilizing tools like IterativeImputer from sklearn.impute. Furthermore, while certain data preprocessing methods can be computationally intensive, judicious selection can help mitigate processing time. Utilizing methods like “RecursiveFeatElim” may be time-consuming, especially with a large feature set. Yet, adopting unsupervised methods for feature reduction can enhance computational efficiency. Additionally, alphaML defaults to a 90% correlation threshold to filter out highly correlated features, which might not suit every user’s needs.

Currently, alphaML incorporates three hyperparameter optimization methods, which primarily focus on key or bespoke parameters, not exhaustive optimization. Despite the potential computational demands of full optimization, certain datasets may necessitate it. The present iteration of alphaML has fixed limits for hyperparameter optimization, designed for ease of use, yet advanced users might desire greater customization. Lastly, our testing encompassed four cancer types, 23 drugs, and six distinct datasets. For broader validation, there is a need for more clinically relevant datasets, which are presently limited in the public domains, as well as diverse practical applications to affirm the platform’s versatility.

In summary, while the alphaML platform is robust in its design, offering a wealth of functionality and adaptability, potential users must be cognizant of certain operational nuances and preset boundaries. From specific data directory placements and file formats to its present label-encoding support and default computational choices, there is a structured path that one must navigate to fully harness its capabilities. Although the platform has been rigorously tested across diverse scenarios, its full potential can be realized further with the availability of more comprehensive, clinically relevant datasets and wider practical applications. Nonetheless, its current configuration underscores a commitment to user-friendliness, computational efficiency, and adaptability to varied data challenges.

## Experimental procedures

### Resource availability

#### Lead contact

For further details or to request resources, please reach out to the primary contact, Julhash U. Kazi at kazi.uddin@med.lu.se.

#### Materials availability

The materials used in this study are publicly available and are detailed in the subsequent data procurement section. Requests for the drug synergy data should be directed to the lead author. Otherwise, the study did not produce any new materials.

#### Data and code availability

The Python scripts are accessible to individuals possessing fundamental computational competencies and rudimentary proficiency in the management of tabular data via applications akin to Microsoft Excel. For enhanced user interaction, we furnish a graphical user interface (GUI). The Python library, alphaML, can be procured from the Python Package Index (PyPI) at www.pypi.org/project/alphaml/, or directly from the GitHub repository at www.github.com/kazilab/alphaML, or from dx.doi.org/10.6084/m9.figshare.24415894.^24^ All data are publicly available and processed data can be downloaded from dx.doi.org/10.6084/m9.figshare.23623077.^9^

### Description of each model

Regularized linear models serve as modifications of the standard linear regression model, integrating a regularization parameter to inhibit overfitting and augment the predictive capacity of the model across diverse datasets. These models are typically formulated to include a penalty term within the loss function, which minimizes both the residuals and the complexity of model.^25^ There are three main types of regularized linear models: ridge, lasso, and elastic net. The ridge model introduces an L2 penalty, which is equal to the square of the coefficient magnitudes scaled by a constant alpha.^26^ This results in the attenuation of coefficient values; however, it does not drive them to zero, thereby not performing feature selection. The least absolute shrinkage and selection operator (lasso) integrates an L1 penalty, which is equivalent to the absolute magnitude of the coefficients.^25^ This type of regularization may induce zero coefficients, thereby performing both parameter shrinkage and feature selection. The elastic net model serves as a hybrid methodology, integrating both L1 and L2 penalties.^27^ It is notably advantageous when multiple features are correlated. By merging aspects from both ridge and lasso, it retains the feature selection capabilities of lasso, while maintaining the regularization properties of ridge. These regularization techniques have been substantiated to be successful across a diverse range of applications, demonstrating both predictive accuracy and model interpretability.

Support vector machines (SVMs) represent a subset of supervised machine learning methodologies utilized for both classification and regression evaluations of datasets. SVMs operate under the theory of structural risk minimization, aiming to attenuate an upper limit on the potential generalization error as opposed to purely minimizing the error identified during training.^28^ The support vector classification (SVC) is a type of SVM used for classification tasks. In its simplest linear form, the algorithm constructs a hyperplane in a high-dimensional feature space to separate the different classes with a maximal margin.^29^ This separating hyperplane is influenced by a subset of the initial training data, referred to as support vectors, which are essentially data points in close proximity to the decision boundary. For nonlinear applications, SVC integrates the kernel trick to implicitly transform the input data into high-dimensional feature spaces, where a linear delineation is sought after. The nu-Support Vector Classification (NuSVC) signifies another SVM variant, where the problem is parameterized with parameters nu, as opposed to the conventional regularization parameter C seen in SVC. The nu parameter (0 < nu ≤ 1) symbolizes an upper limit on the training errors proportion, and a lower limit on the fraction of support vectors.^30^ The NuSVC model provides a way of controlling the number of support vectors and margin errors, thus offering a nuanced control over the model complexity. Both SVC and NuSVC are effective in high-dimensional spaces, even when the number of dimensions exceeds the number of samples, and are versatile in their choice of decision function, which can be linear, polynomial, or a radial basis function.

Ensemble methods combine the predictions of multiple models to create a final prediction that is more accurate and robust than the individual models alone. The theoretical foundation of these methods is predicated on the amalgamation of weak learners to create a robust learner. The classification of ensemble methods can largely be bifurcated into two principal types: boosting and bagging. Boosting is an ensemble strategy that is designed to evolve a robust classifier from a series of weak classifiers. This is accomplished by building a model predicated on the training data, followed by the creation of a subsequent model that aims to rectify the errors manifested by the former model. Model addition continues either until the training set is flawlessly predicted or until a predefined threshold of models is reached. Adaptive boosting (AdaBoost) is one of the pioneering boosting algorithms that operates by assigning weights to the observations, attributing higher weightage to challenging instances to classify, and lower to those instances that are classified efficiently.^31^ Gradient boosting was later proposed, which endeavors to correct the residual errors of the previous predictor by employing gradient descent to minimize the loss when new models are incorporated.^32^ Histogram-based gradient boosting (HistGradientBoosting) is a variant of gradient boosting that discretizes continuous input features into integer-valued bins, leading to expedited computation and reduced memory footprint.^33^ Extreme gradient boosting (XGBoost) is a robust and scalable implementation of gradient boosting algorithms, esteemed for its execution velocity and model performance.^34^ Microsoft Corporation developed the LightGBM, a gradient boosting framework known for its distributed and efficient architecture, superior training speed, efficiency, and capability to manage large-scale data.^35^ CatBoost, another gradient boosting algorithm, displays exceptional prowess in managing categorical variables.^36^ In contrast to boosting algorithms, bagging (an acronym for bootstrap aggregating) is an ensemble method that involves manipulating the training set through resampling and executing algorithms on it. Bagging diminishes the variance of a base estimator (e.g., a decision tree), by incorporating randomization into its construction procedure and subsequently creating an ensemble from it. The bagging algorithm, Random Forest, merges the concept of bagging and the method of random subspace to fabricate a series of decision trees with regulated variance.^15^ The random forest variant extremely randomized trees (Extra Trees) utilizes a random subset of features to bifurcate the data at each node during the construction of the trees.^37^

Artificial neural networks (ANNs) are intricate structures composed of interconnected layers of computational units, often referred to as "neurons." These units are responsible for the conversion of input data into corresponding outputs. Inter-neuron communication is enabled through connections that facilitate the transfer of signals from one neuron to another. Upon receipt, the signal undergoes processing by the recipient neuron, which subsequently transmits signals to successive neurons in the connection chain. This architecture is hierarchically layered, commencing with an input layer where raw data are introduced. The data then traverses one or more intermediary or "hidden" layers, each responsible for performing specific data transformations, before culminating at the output layer. The MLPC is a subset of the broader category of feedforward ANNs.^38^ MLPs are characterized by a structure comprising a minimum of three node layers: an input layer, a hidden layer, and an output layer. With the exception of the input nodes, each node functions as a neuron employing a nonlinear activation function. For training, MLPs deploy a supervised learning methodology known as backpropagation.^38^ Last, the TabNet algorithm introduces an innovative, high-performance, and interpretable neural network model tailored for tabular data. This model’s distinctive feature is its sequential attention mechanism, which enables the selection of pertinent features at each decision juncture, enhancing its interpretability compared with conventional feedforward networks or tree-based models. This sequential attention mechanism operates as a learnable mask deployed at each decision point, thus empowering the model to choose the relevant features to examine for each decision.^39^

### NegLog2RMSL

The NegLog2RMSL is a composite metric that amalgamates seven distinct metrics: Hamming loss (HL), negative likelihood ratio (NLR), false negative rate (FNR), false discovery rate (FDR), false omission rate (FOR), false positive rate (FPR), and Brier score loss (BSL). The rationale behind the construction of NegLog2RMSL has been elucidated in prior work.^7^ In brief, when we assess the performance of a model, using multiple performance metrics is beneficial as it offers a holistic evaluation, encompassing various facets of prediction quality. A single metric might only provide a snapshot of the model’s capability, but integrating several metrics paints a more detailed picture. Specifically, the NegLog2RMSL metric, derived from the geometric mean of the root-mean-square values (both diff and loss), ensures that the evaluation is nuanced. It captures the tendencies of overfitting and underfitting, as well as pinpointing other potential biases in a model’s performance. Furthermore, the transition to a negative log2 scale bestows a standardized scaling on the metric. This uniformity facilitates easier comparisons and interpretations when evaluating different models or when considering various datasets. The beauty of the NegLog2RMSL metric lies in its design—it was crafted with clarity in mind. By offering a singular value that encapsulates a range of performance dimensions, it streamlines the often complex process of comparing and selecting the most apt model. To construct the metric, we commence by computing individual metrics for both the training and test/validation samples. The difference between the training and test scores for each metric is then captured by the formula:

$D=\sqrt{\frac{1}{n}\sum_{j=1}^{n}{\left(M_{j}^{train}-M_{j}^{test}\right)}^{2}}$ , where *j* indexes the specific metrics mentioned above.

Simultaneously, we calculate the test loss using

$$L=\sqrt{\frac{1}{n}\sum_{j=1}^{n}{\left(M_{i}^{test}\right)}^{2}}$$

Ultimately, the NegLog2RMSL is determined by

$${\text{NegLog}2\text{RMSL}=-\log}_{2}\sqrt{\left(D+0.05\right)\times L}$$

This formulation has been adapted from a previously described methodology.^7^

### Custom scores for hyperparameter tuning

The methodology employed for hyperparameter tuning incorporated a compound scoring system that utilized both Cohen’s kappa coefficient and the Matthew’s correlation coefficient (MCC).^7^ This amalgamated scoring strategy ensures a rigorous evaluation of categorization models by harnessing the unique capabilities inherent in each metric. Cohen’s kappa, with its numerical values spanning from −1 to 1, quantifies the concurrence between two independent classifiers, effectively controlling for random chance and imbalance in class distribution. Conversely, the MCC, possessing an identical range, appraises binary categorization systems by contemplating all constituents of the confusion matrix, demonstrating resilience toward class disequilibrium. This dualistic metric strategy, exhibiting reduced sensitivity to class distribution skewness and offering an exhaustive appraisal of accuracy, fosters dependable evaluation of classifier efficacy, thus supporting superior model selection for pragmatic implementation. We employed the geometric mean of kappa and MCC errors, notated as "kappa_mcc_error" (KME), and computed using the following formula.

$$\text{KME}=1-\sqrt{Kappa\times MCC}$$

This method was also described previously.^7^ Alongside this, the "kappa_mcc_error" was utilized in conjunction with a controlled fitting approach, implemented to mitigate both overfitting and underfitting tendencies in our model. We used a "custom_score", which incorporates the "kappa_mcc_error" in a bipartite manner, taking into account both training and validation scores. To derive the "custom_score," we computed the "kappa_mcc_error" for both training (KME^train^) and testing/validation (KME^test^) samples. Following this, we applied the following formula:

$$\text{custom}\_\text{score}=\sqrt{{KME}^{test}\times |{KME}^{train}-{KME}^{test}|}$$

This custom score seeks to minimize the discrepancy between training and testing errors, guiding the selection of hyperparameters to counteract overfitting or underfitting.

### Data procurement

The source of annotated data was derived from the scDEAL and BeatAML research studies.^40^^,^^41^ The dataset utilized for attribute selection was procured from The Cancer Genome Atlas (TCGA) and the Therapeutically Applicable Research to Generate Effective Treatments (TARGET) databases. The AML data employed for prognostic analyses was obtained from the GDC data portal (portal.gdc.cancer.gov). The credit card fraud detection data (www.kaggle.com/datasets/mlg-ulb/creditcardfraud) and Pima Indians diabetes database (www.kaggle.com/datasets/uciml/pima-indians-diabetes-database) were collected from Kaggle. The bank marketing data (archive.ics.uci.edu/dataset/222/bank+marketing), NATICUSdroid (Android Permissions) dataset (archive.ics.uci.edu/dataset/722/naticusdroid+android+permissions+ dataset), predict students' dropout and academic success dataset (archive.ics.uci.edu/dataset/697/predict+students+ dropout+and+academic+success), and Breast Cancer Wisconsin (diagnostic) dataset (archive.ics.uci.edu/dataset/17/breast+cancer+wisconsin+diagnostic) were collected from the University of California Irvine Machine Learning Repository.

## Supplemental information

Document S1. Figures S1–S3 and Note S1

Document S2. Article plus supplemental information
