# Supplementary material for: AlphaML: A clear, legible, explainable, transparent, and elucidative binary classification platform for tabular data
Source: Patterns (N Y). 2023 Dec 13;5(1):100897. doi: 10.1016/j.patter.2023.100897 (PMC10801203; doi:10.1016/j.patter.2023.100897)
Supplement: Document S1. Figures S1–S3 and Note S1 [file mmc1.pdf]

Patterns, Volume *5*

Supplemental information

AlphaML: A clear, legible, explainable, transparent, and elucidative binary classification platform for tabular data

Ahmad Nasimian, Saleena Younus, Özge Tatli, Emma U. Hammarlund, Kenneth J. Pienta, Lars Rönnstrand, and Julhash U. Kazi

Supplemental Information

Supplemental figures

Figure S1. SHAP plots illustrate the contribution of each feature to the model's global prediction. The Breast Cancer Wisconsin (Diagnostic) dataset was used to calculate global SHAP scores. These plots provide insight into the relative global importance of features.

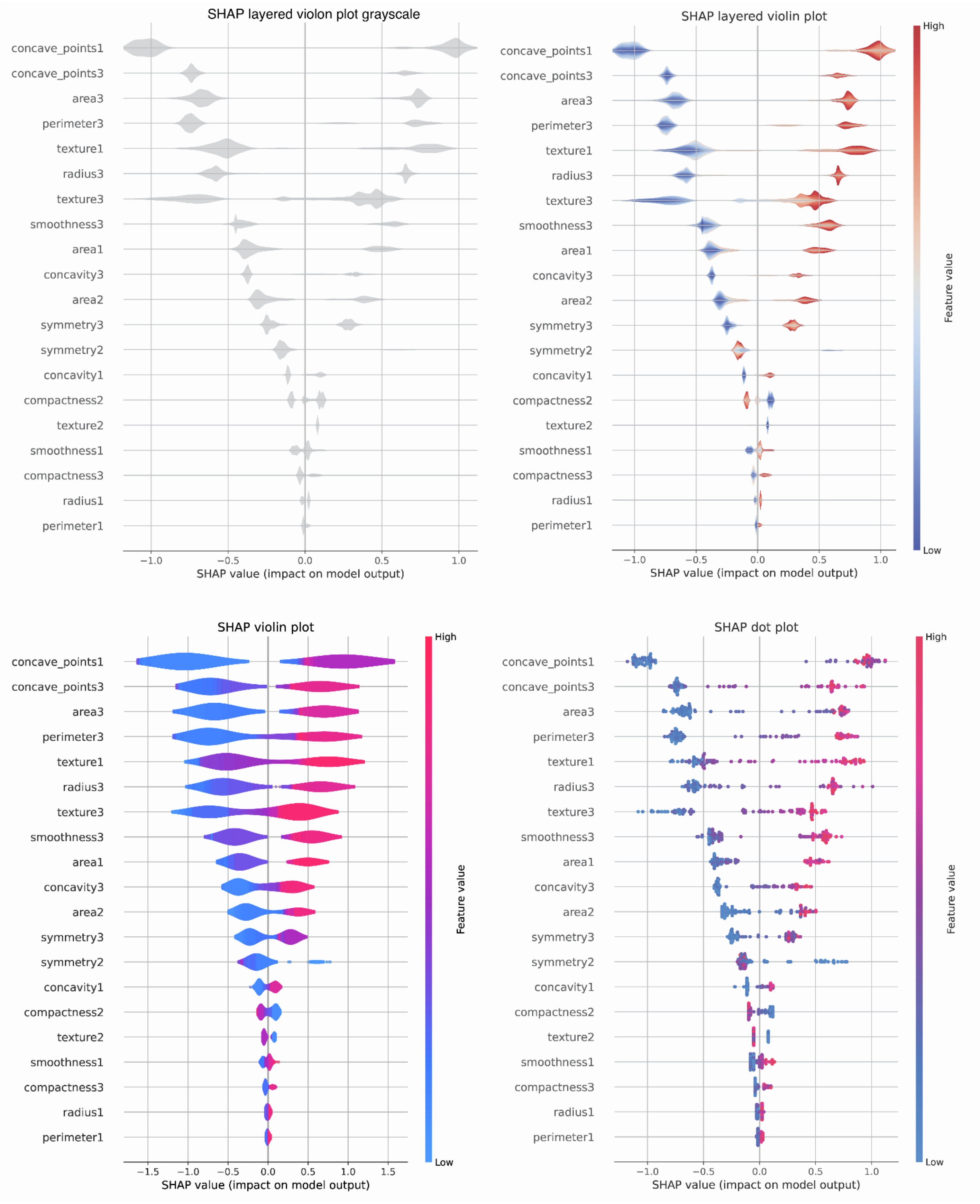

Figure S2. SHAP plots illustrate the contribution of each feature to the model's local prediction. The Breast Cancer Wisconsin (Diagnostic) dataset was used to calculate local SHAP scores. These plots provide the importance of features and their impact on the prediction outcomes (the first eight examples are appended).

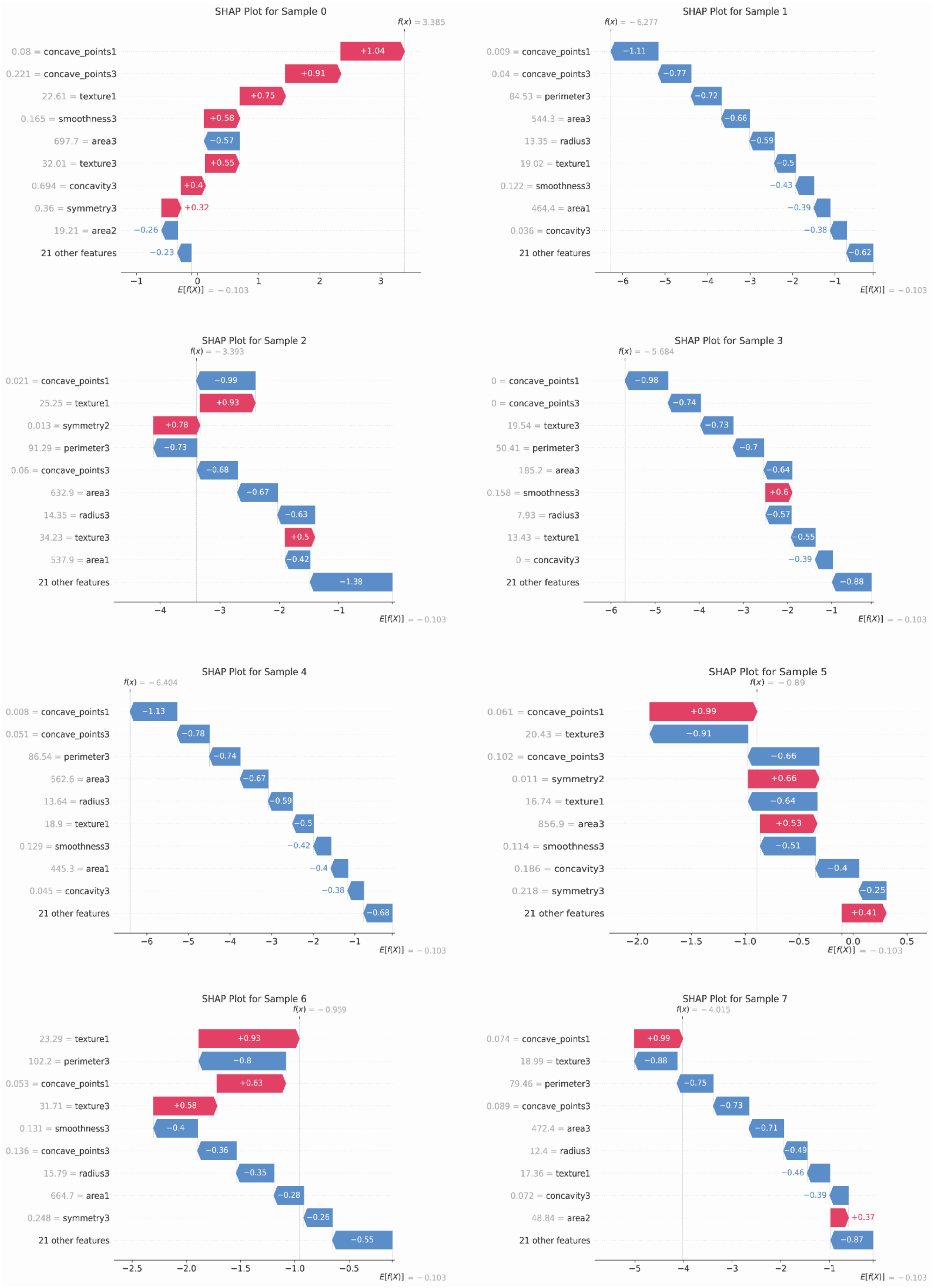

Figure S3. LIME plots showcase the local interpretation of the model's decisions for individual predictions. The Breast Cancer Wisconsin (Diagnostic) dataset was used to calculate LIME scores. The LIME plots elucidate how each feature influences specific predictions, ensuring transparency in the model's decision-making process (The first eight examples are appended).

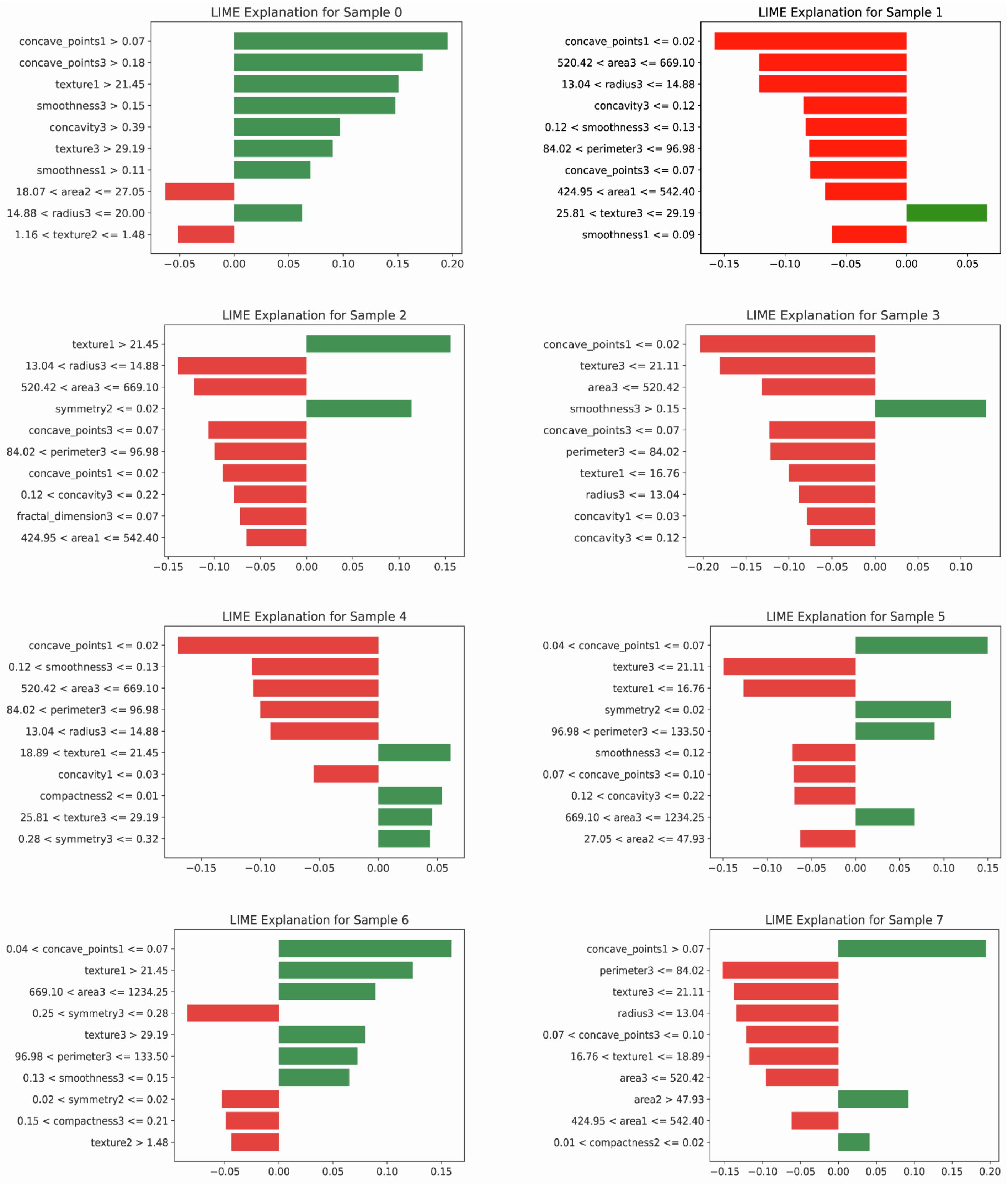

Supplemental Note

Note S1. Procedure for configuring the alphaML platform within a Python-based computational environment.

Installation:

1. *Install Anaconda*: Download the latest version of Anaconda from www.anaconda.com. Install by double-clicking the downloaded installer. When the installer asks for the installation type, choose "Just Me".
2. *Install alphaml*: For Windows - search for *Anaconda Powershell Prompt in Windows search and click on it*. Type the following command and press "Enter" to install alphaml and dependent packages.
   `pip install alphaml`
   For Mac - search for Terminal and click on it. Before installing the package, activate the base conda environment by typing the following command and pressing "Enter":
   `conda activate`
   After seeing "base" prepended to the prompt, type the following command and press "Enter" to install alphaml:
   `pip install alphaml`
   After installation, deactivate the conda environment by the following command:
   `conda deactivate`
   Alternatively, Anaconda Navigator can be used (platform independent). Launch Anaconda Navigator and from the navigator launch JupyterLab. It will open a browser window. Click on Terminal from the browser window. Type the following command in the Terminal/PowerShell window and press "Enter" to install alphaml:
   `pip install alphaml`

Alternative installation (platform independent):

After installing Anaconda, launch "**JupyterLab**" by clicking "*Launch*" in the Anaconda Navigator. This action will open a browser window. In this window, select "*Terminal*" to open a new terminal tab within the JupyterLab interface. Once the terminal is open, enter the command "`pip install alphaml`" to install alphaml.

Run alphaml:

1. In Windows, search for "Anaconda Powershell Prompt" in the Windows search bar and click on it. Type the following command and press "Enter" to open the alphaML GUI:
   `python -c "from alphaml import guir"`
2. In Mac, open Terminal. First, activate the base conda environment with the following command:
   `conda activate`
   After seeing "base" prepended to the prompt, run the alphaML GUI with the following command:
   `python -c "from alphaml import guir"`
   After use, deactivate the conda environment by typing the following command:

   `conda deactivate`

3. If Anaconda Navigator is being used, launch it and then launch JupyterLab from within Navigator. This will open a browser window. Click on "Terminal" in the browser window, then type the following command and press "Enter" to open the alphaML GUI:

   `python -c "from alphaml import guir"`

Alternative (platform independent):

From the Anaconda Navigator, launch "JupyterLab" by clicking on "Launch." Once in the JupyterLab window, click on "Terminal" to open the Terminal window. In the Terminal, type "`python -c "from alphaml import guir`" and then press "Enter".

Using alphaML from the GUI:

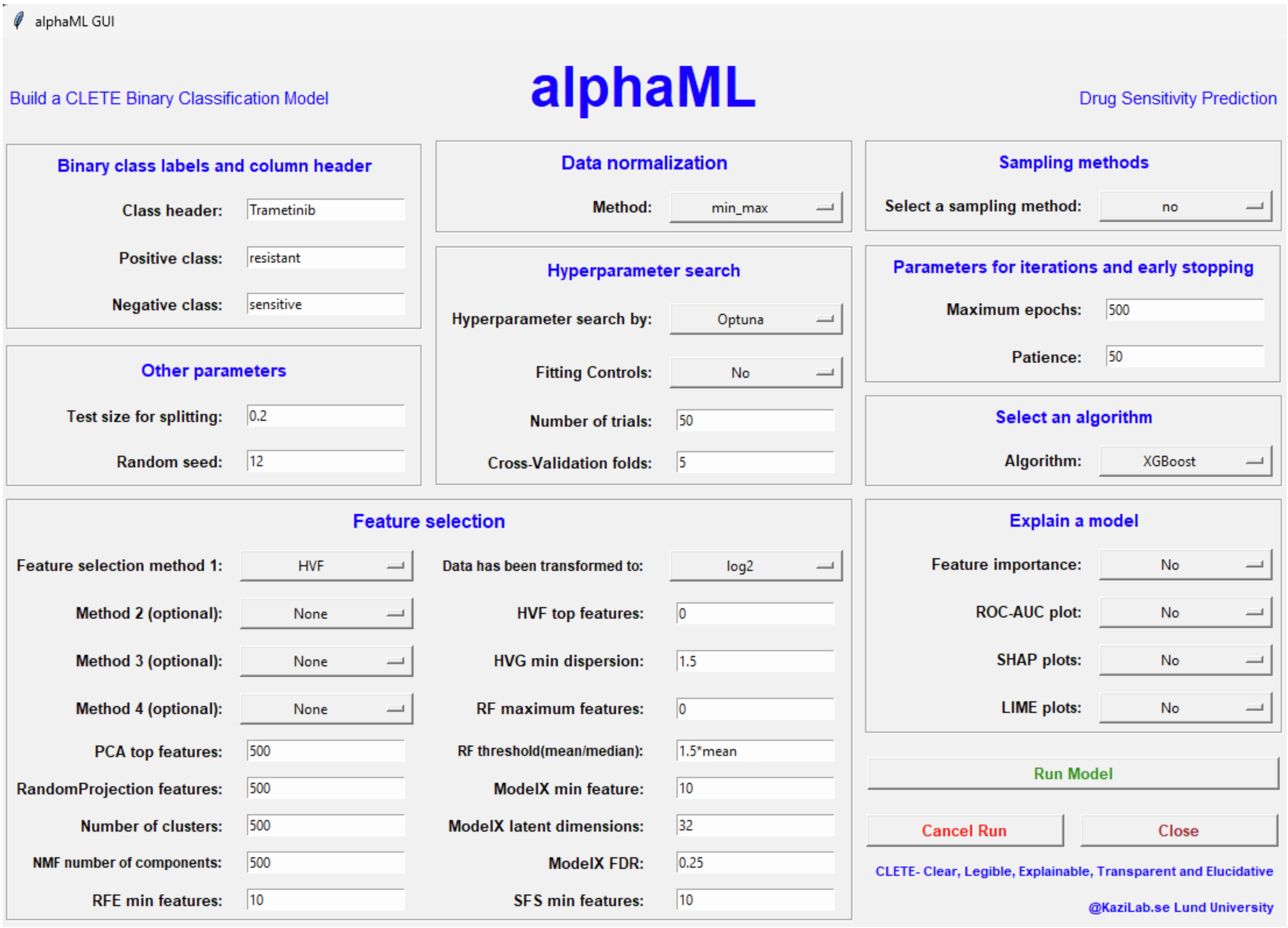

1. Launch the alphaML GUI. Press the "Run Model" button. This will automatically generate two folders, namely "alphaML_data" and "alphaML_results", in the current user's Documents directory.
2. The "alphaML_data" folder should contain at least five files named "data_for_feature_selection.csv", "data_labels.csv", "labeled_data.csv", "suggested_features.csv", and "unlabeled_data.csv". alphaML first checks the presence of these files and, if they are missing, it will attempt to download sample data. Please ensure that these filenames are unaltered, as they have been hardcoded into alphaML.
3. The "labeled_data.csv", "data_for_feature_selection.csv", and "unlabeled_data.csv" files should all contain tabular data. The first column of these tables should include sample names, and each row must represent a distinct sample. Ensure the column headers denote feature names. The "data_labels.csv" file should contain binary labels corresponding to the labeled data. The "suggested_features.csv" file, a single-column file, is designed to accept pre-selected features for model input.
   Please note: The "labeled_data.csv" file is considered as the annotated data which has corresponding binary labels in the "data_labels.csv".

   Example: "labeled_data.csv".

Sample	A1BG	A2M	A2ML1	A4GALT	AAAS	AACS	AADAT
DATA.683(	3.803976	3.650142	2.897336	3.176677	5.034564	3.923051	3.744538
DATA.683(	5.128699	3.607324	2.812968	3.301372	4.664793	4.079043	3.624393
DATA.684(	4.251872	3.3336	4.494838	3.45911	5.706589	3.832364	4.675207

Example: "data_labels.csv "

Sample	Afatinib	Alisertib	Axitinib	Bms34554	Bortezom	Cediranib	Crizotinib
DATA.683	resistant	sensitive	resistant	resistant	sensitive	resistant	resistant
DATA.683	resistant	resistant	resistant	sensitive	sensitive	resistant	resistant
DATA.684	resistant	resistant	resistant	resistant	resistant	resistant	resistant

The "data_for_feature_selection.csv" file should contain the data you intend to use for feature selection. This can include specific data related to a particular disease. If you don't have specific data for this purpose, you can use your labeled data. In this case, copy the contents of your "labeled_data.csv" file into the "data_for_feature_selection.csv" file.

Example: " data_for_feature_selection.csv"

Sample	A1BG	A2M	A2ML1	A4GALT	AAAS	AACS	AADAT
TARGET-1	0.701416	0	0.007913	0.076012	2.145677	1.685761	0.415651
TARGET-1	0.976877	0.159758	0.009204	0.067088	3.704739	2.545029	2.791981
TARGET-1	0.340961	0.0397	0.008774	0.021622	3.684965	0.700528	1.010851

The "unlabeled_data.csv" file should contain the unannotated data, which lacks binary labels. If you don't have a separate set of unannotated data, you can use your labeled data. In this case, copy the contents of your "labeled_data.csv" file into the "unlabeled_data.csv" file.

Example: "unlabeled_data.csv"

	A1BG	A2M	A2ML1	A4GALT	AAAS	AACS	AADAT
aml_ohsu_	0.330713	0.280005	0.02153	0.520474	5.475134	2.719014	0.284122
aml_ohsu_	0.333274	1.813633	0.05036	4.993026	5.252081	2.81072	0.212554
aml_ohsu_	0.310227	0.609844	0.087837	3.126578	5.579161	2.789831	0.192626
aml_ohsu_	0.029552	2.409636	0.08932	1.253376	5.486577	3.227344	1.1814

4. *Binary class labels and column header:* The "Class header" is used to find the input string in data_label.csv for information about the binary classes. Input in the "Class header" must be identical to the respective class header in "data_labels.csv". The "data_labels.csv" requires at least two columns: "Sample" and a column with class information.
The "Positive class" represents the outcome or event of interest for prediction, such as resistance, cancer, diabetes, etc. Conversely, the "Negative class" represents the other possible outcome or event, such as sensitivity, healthy control, etc. The binary class label converter in alphaML removes empty spaces and converts labels to lowercase before encoding the "Positive class" label as "1" and the "Negative Class" label as "0". For instance, "downregulated" or "Downregulated" or "Down regulated" or "Down Regulated" will all be encoded as the same class.

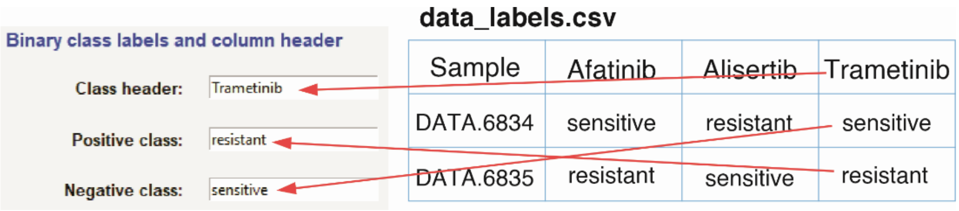

5. *Other parameters:* The parameter "Test size for splitting" determines the division of samples for model building and testing. By default, the ratio of training to test samples is 80:20, represented by 0.2 (test sample size). Modify this parameter between 0.05 and 0.5 to alter the test sample size. The "Random seed" should be a positive number.
6. *Feature selection module:* This module extracts important features or loads predefined features for model development. The feature selection module in alphaML provides access to a panel of feature selection methods. Users can use a single feature selection method from the drop-down list or use up to four methods in combination. Method 1 is mandator and from methods 2, 3, and 4, the user can select any or none of them.

PCA top features: Used by SelByPCA (select by PCA) method and can be between 1 and the initial number of features.

Random Projection features: Used by RandomProjection method and can be between 1 and the initial number of features.

Number of clusters: Used by SelByClustering (select by clustering) method and can be between 1 and the initial number of features.

NMF number of components: Used by SelByNMF (select by NMF) method and can be between 1 and the initial number of features.

RFE number of components: Used by RecursiveFeatElim (Recursive feature elimination) method and can be between 1 and the initial number of features. Computationally expensive method and is not recommended for a large number of initial features.

Data has been transformed to: To be used by the HVF (highly variable features) method. HVF converts log-transformed data to anti-log, therefore, it is important to mention here what type of log transformation has been used in the data appended in "data_for_feature_selection.csv" file. Select from a drop-down list. Select None if data has not been transformed to log.

HVF top features: Used by HVF (highly variable features). The number of features to be selected. Input can be between 1 and the initial number of features. If "0", this option will be ignored.

HVF Min dispersion: Used by HVF (highly variable features). The number of features that pass normalized dispersion value. Input can be any positive number. Only used if *HVF top features* are "0", otherwise, this option will be ignored.

RF Maximum features: Used by SelectByRF (select by random forest). The maximum number of features to be selected that pass the "RF threshold". If "0", this option will be ignored.

RF Threshold: Used by SelectByRF (selected by random forest). To select features that pass a threshold. It can be mean or median and multiplied by a positive number.

ModelX Min features: Used by ModelX (Model-X knockoffs). The suggested minimum number that should be passed to proceed with the selected features. If ModelX identifies fewer features than the "*ModelX Min features"* the alphaML feature selection module will ignore this method and continue with the input features.

ModelX Latent dimension: Used by ModelX (Model-X knockoffs). Latent dimensions to be used by autoencoder incorporated in ModelX method.

ModelX FDR: Used by ModelX (Model-X knockoffs). False discover rate cut-off to be used by ModelX.

SFS Min features: Used by SeqFeatSel (Sequential Feature selection) method and can be between 1 and the initial number of features. Computationally expensive method and is not recommended for a large number of initial features.

The "RemoveHighCorrFeat" method in Method 3 and Method 4 can be applied to remove the feature that correlates more than 90% with another feature.

The "IterativeFeatSel" method in Method 3 and Method 4 can be applied to select 1 to 3 most important features. Computationally extremely expensive for a large number of initial features.

The "None" method in Method 2, Method 3, and Method 4 can be used to ignore a method.

Feature selection module can be used independently by selecting. "

7. Data Normalization: Two data normalization options are available: "min_max" and "standardization". If no normalization is required, select "None" from the dropdown list.
8. *Hyperparameter search:* Optuna, Bayes, or Grid search methods can be selected from the dropdown menu. These represent Optuna, BayesSearchCV, and GridSearchCV respectively. A "Predefined" option, where all parameters are hardcoded, is also available.

   Fitting Controls: This option is applicable only for "Optuna". From a drop-down menu if *"Yes"* is selected, Optuna will use a custom scoring method that considers train metrics to control overfitting.

   Number of trials: How many trials are to be performed for hyperparameter search?

   Cross-validation folds: Number of cross-validations to divide data.
9. *Sampling methods:* By default, alphaML does not apply any sampling method. However, "under" and "over" sampling methods can be selected from the dropdown menu.
10. *Parameters for iterations and early stopping:* Used by TabNet only.
11. *Select an algorithm:* An algorithm can be chosen from 15 different options available in a dropdown menu. Alternatively, a quick test can be performed by selecting "Test_Briefly". If "None" is selected, no model will be built.
12. Explain a model: To perform any or all of four methods, including "Feature importance", "ROC-AUC plot", "SHAP plots", and "LIME plots", select from dropdown menus.
13. To initiate the run, click on the "Run Model" button. Click on the "Cancel Run" button to abort a run and the "Close" button to close the GUI window.

The alphaML result files:

1. Depending on how many feature selection methods have been selected, the result folder can contain 1-4 CSV files with names as follows:
   ClassHeader_Method1_None_ None _ None_Feat_M1.csv
   ClassHeader_Method1_Method2_ None_ None_Feat_M2.csv
   ClassHeader_Method1_Method2_Method3_ None_Feat_M3.csv
   ClassHeader_Method1_Method2_Method3_Method4_Feat_M4.csv
2. Two CSV files containing normalized labeled and unlabeled data.
   ClassHeader_Method1_normalized_labeled_data.csv
   ClassHeader_Method1_normalized_unlabeled_data.csv
3. A PDF file containing parameters identified by hyperparameter search.
   ClassHeader_SamplingMethod_HyperparameterSearch_Algorithm_fit_FittingControl_parameters.pdf
4. The model file that can be used for prediction.
   ClassHeader_SamplingMethod_HyperparameterSearch_Algorithm_fit_FittingControl_model.pkl
5. An accuracy and loss plot showing both training and test scores.
   ClassHeader_SamplingMethod_HyperparameterSearch_Algorithm_fit_FittingControl_valid_accuracy_curve.pdf
6. A PDF file containing the confusion matrix will be created using test samples.
   ClassHeader_SamplingMethod_HyperparameterSearch_Algorithm_fit_FittingControl_confusion_matrix.pdf
7. A CSV file containing train and test scores.
   ClassHeader_SamplingMethod_HyperparameterSearch_Algorithm_fit_FittingControl_scores.csv
8. An XLSX file containing true test labels, prediction labels, and prediction probability.
   ClassHeader_SamplingMethod_HyperparameterSearch_Algorithm_fit_FittingControl_ test_prediction.xlsx
9. A CSV file containing feature importance calculated by the model (if available) and permutation importance.
   ClassHeader_SamplingMethod_HyperparameterSearch_Algorithm_fit_FittingControl_global_feat_imp.csv
10. A PDF file containing global SHAP plots and SHAP plots for each test sample.
    ClassHeader_SamplingMethod_HyperparameterSearch_Algorithm_fit_FittingControl_ test_SHAP.pdf
11. A PDF file containing LIME plots for each test sample.
    ClassHeader_SamplingMethod_HyperparameterSearch_Algorithm_fit_FittingControl_ test_LIME.pdf
12. A CSV file containing avu, fpr, tpr, accuracy, and thresholds for repeated k-fold analysis.
    ClassHeader_SamplingMethod_HyperparameterSearch_Algorithm_fit_FittingControl_repeated_accuracy.csv
13. A PDF file containing the ROC-AUC curve from the repeated k-fold analysis.
    ClassHeader_SamplingMethod_HyperparameterSearch_Algorithm_fit_FittingControl_ AUC_curve.pdf
14. A PDF file containing a dot plot of accuracy measurements from repeated k-fold analysis.
    ClassHeader_SamplingMethod_HyperparameterSearch_Algorithm_fit_FittingControl_accuracy_plot_k_fold_cv_roc.pdf
15. A text file containing a log.
    ClassHeader_SamplingMethod_HyperparameterSearch_Algorithm_fit_FittingControl_alphaML_run.log
16. If Optuna is used for hyperparameter search, an additional PDF file with hyperparameter importance will be created.
    ClassHeader_SamplingMethod_HyperparameterSearch_Algorithm_fit_FittingControl_optuna_param_importance.pdf

Run a prediction using alphaPred:

1. In Windows, search for *Anaconda Powershell Prompt in Windows search and click on it*. Type the following command and press "Enter" to open the alphaPred GUI.
   ```
   python -c "from alphaml import guip"
   ```
2. In Mac, search for Terminal, click on it, type the following command, and press "Enter".
   ```
   conda activate
   ```
   “base” should be prepended, then type the following command and press "Enter" to open alphaPred GUI.
   ```
   python -c "from alphaml import guip"
   ```

   After use deactivate the conda environment by the following command.

   ```
   conda deactivate
   ```

3. If Anaconda Navigator is being used, Launch Anaconda Navigator, and from the navigator Launch JupyterLab. It will open a browser window. Click on Terminal from the browser window. Type the following command in the Terminal/PowerShell window and press "Enter" to open alphaPred GUI.

```
python -c "from alphaml import guip"
```

Using alphaPred from the GUI:

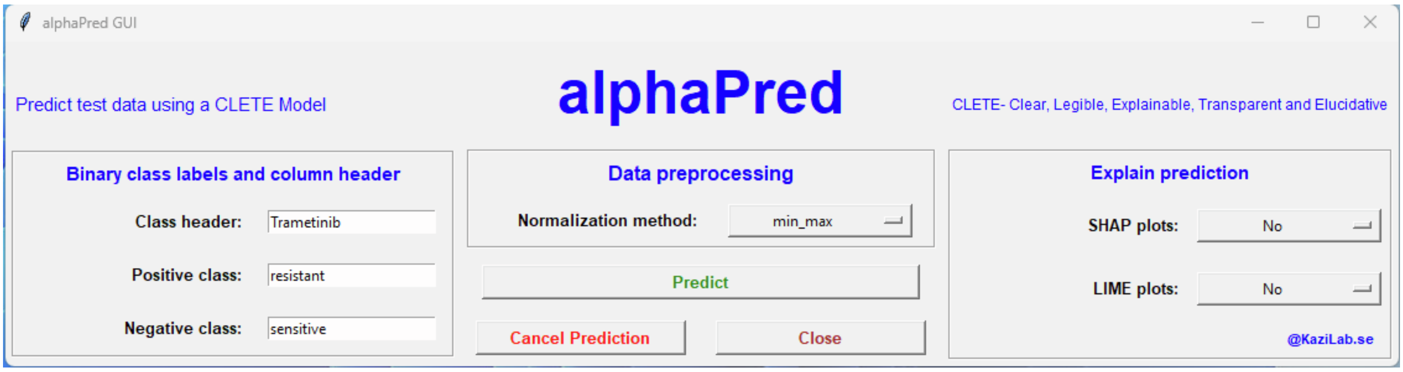

1. Once alphaPred GUI is opened, press on "Predict". The alphaPred will create two folders (alphaPred_data and alphaPred_results) in the current user's Documents folder.
2. The alphaML_data folder should contain at least three files with the names "model.pkl", "selected_features.csv", and "test_data.csv". The alphaPred first checks the availability of these three files, otherwise, attempts to download example data. Names of all three files have been hard coded in alphaPred, so must be kept the same.
3. The "model.pkl" is the model file built by alphaML to be used for prediction. The model file should be copied from alphaML_results and renamed.
4. The "selected_features.csv" contains features used to build the model. This file should be copied from alphaML_results and renamed.

   Example: "selected_features.csv"

Highly Var
ABCA13
ABCA2
ABTB2
ACOT11

5. The "test_data.csv" contains tabular data for prediction. The first column should contain sample names and each row should represent a sample. Column headers must represent feature names.

   Example: "test_data.csv".

Sample	A1BG	A2M	A2ML1	A4GALT	AAAS	AACS	AADAT
TARGET-1(	0.701416	0	0.007913	0.076012	2.145677	1.685761	0.415651
TARGET-1(	0.976877	0.159758	0.009204	0.067088	3.704739	2.545029	2.791981
TARGET-1(	0.340961	0.0397	0.008774	0.021622	3.684965	0.700528	1.010851
TARGET-1(	0.327687	0.050467	0	0.036609	3.746033	1.06757	0.122871
TARGET-1(	0.201885	0.3744	0.033511	0.1595	1.239398	0.983969	2.133892

6. *Binary class labels and column header:* The "Class header" does not have any role in prediction but can be useful for documentation. The "Positive class" and "Negative class" names are used to convert "1" and "0" to class labels.
7. *Data preprocessing:* The same preprocessing method must be applied that is used for alphaML model building.
8. *Explain prediction:* SHAP plots and LIME plots can be generated by selecting "Yes" from the drop-down menu.
9. Clicking on the "Predict" button will initiate the prediction. The "Cancel Run" button will abort a run and click on the "Close" button to close the GUI window.

The alphaPred result files:

1. A CSV file containing normalized test data.
   ClassHeader__Algorithm_fit_normalized_test_data.csv
2. An XLSX file containing sample name, prediction class, and probability scores.
   ClassHeader__Algorithm_fit__test_prediction.xlsx
3. Two PDF files with SHAP and LIME plots.
   ClassHeader__Algorithm_fit__test_SHAP.pdf
   ClassHeader__Algorithm_fit__test_LIME.pdf
4. A text file containing a log.
   alphaPred_run.log

Known Shortcomings:

On the Mac OS, the launch of the alphaml GUI might fail if an older version of "lightgbm" and/or "imbalanced-learn" exists in the Conda environment. To resolve this, please install "lightgbm" using "`conda install lightgbm`" and "imbalanced-learn" using "`pip install imbalanced-learn`".
